# Radiomics Based on Multiparametric Magnetic Resonance Imaging to Predict Extraprostatic Extension of Prostate Cancer

**DOI:** 10.3389/fonc.2020.00940

**Published:** 2020-06-16

**Authors:** Lili Xu, Gumuyang Zhang, Lun Zhao, Li Mao, Xiuli Li, Weigang Yan, Yu Xiao, Jing Lei, Hao Sun, Zhengyu Jin

**Affiliations:** ^1^Department of Radiology, Peking Union Medical College Hospital, Peking Union Medical College, Chinese Academy of Medical Sciences, Beijing, China; ^2^Deepwise AI Lab, Deepwise Inc., Beijing, China; ^3^Department of Urology, Peking Union Medical College Hospital, Peking Union Medical College, Chinese Academy of Medical Sciences, Beijing, China; ^4^Department of Pathology, Peking Union Medical College Hospital, Peking Union Medical College, Chinese Academy of Medical Sciences, Beijing, China

**Keywords:** radiomics, magnetic resonance imaging, prostate cancer, neoplasm staging, extraprostatic extension

## Abstract

**Background:** To develop a radiomics model based on multiparametric MRI (mpMRI) for preoperative prediction of extraprostatic extension (EPE) in patients with prostate cancer (PCa).

**Methods:** Ninety-five pathology-confirmed PCa patients with 115 lesions (49 positive and 66 negative) were retrospectively enrolled. A 3.0T MR scanner was used to perform T2-weighted imaging (T2WI), diffusion-weighted imaging (DWI), and dynamic contrast-enhanced imaging (DCE). Radiomics features extracted from T2WI, DWI, apparent diffusion coefficient (ADC), and DCE were used to build a radiomics model. Patients' clinical and pathological variables were also obtained to build a clinical model. The radiomics model and clinical model were further integrated to build a combined nomogram. All lesions were randomly divided into the training group (82 lesions) and the validation group (33 lesions). A least absolute shrinkage and selection operator (LASSO) regression algorithm was applied to build the radiomics model. The diagnostic performance of different models was assessed by calculating the area under the curve (AUC) and compared using the Delong test. The calibration curve and decision curve analyses were used to assess the calibration and clinical usefulness of the radiomics model.

**Results:** The AUC values for the radiomics model in the training and validation group were 0.919 and 0.865, respectively, with a good calibration performance. The decision curve analysis confirmed the clinical utility of the radiomics model. The accuracy, sensitivity, and specificity were 81.8, 71.4, and 89.5% in the validation group. In the validation group, the radiomics model outperformed the clinical model (AUC = 0.658, *P* = 0.020), and was comparable with the combined nomogram (AUC = 0.857, *P* = 0.644).

**Conclusion:** The radiomics model based on mpMRI could different EPE and non-EPE lesions with satisfactory diagnostic performance, and this model might assist in predicting EPE before prostatectomy.

## Introduction

Prostate cancer (PCa) is the most common malignancy in men worldwide and also the second leading cause of cancer-related death (1). Additionally, the incidence of PCa has significantly increased in recent decades (2). Studies have shown that the presence of extraprostatic extension (EPE) in radical prostatectomy (RP) specimens was highly predictive of death from prostate cancer (3) and indicated a higher risk of biochemical recurrence (4). The preoperative prediction of EPE has a profound impact on treatment decision making. Patients without EPE could consider RP or active surveillance according to their risk stratification; however, patients with EPE are recommended to undergo nerve-sacrificing RP or adjuvant radiotherapy (5).

Clinical models [such as Partin tables, the Cancer of the Prostate Risk Assessment (CAPRA) score and the Memorial Sloan Kettering Cancer Center nomogram] based on clinical and histopathological variables have been developed to predict EPE. Nevertheless, the diagnostic performances of these models are unsatisfactory with reported areas under the curve (AUCs) ranging from 0.702 to 0.806 (6, 7), and their clinical utility is limited. The preoperative accurate diagnosis of EPE remains challenging (7, 8).

Multiparametric magnetic resonance imaging (mpMRI) has emerged as an important tool in the diagnosis and local staging of prostate cancer (9, 10). However, regarding the diagnosis of EPE, the sensitivity of subjective mpMRI evaluation is insufficient, with a reported sensitivity of 0.57 (95% confidence interval [CI]: 0.49–0.64), and specificity of 0.91 (95% CI: 0.88–0.93) (11). Additionally, the accuracy is strongly correlated with the experience of radiologists (12). Radiomics is defined as high-throughput extraction of mineable, quantitative, and high-dimensional medical imaging features using machine learning (13, 14). Recently, the emerging radiomics technique has been widely applied in PCa research, which was reported to have added value in PCa detection, aggressiveness assessment, and survival analysis (15–20). One of the explanations of radiomics' superiority maybe that radiomics could provide more information about the lesion which might be correlated with the intratumor heterogeneity (13). This quantitative method was also demonstrated to be useful for EPE prediction (6, 21), but only T2-weighted images were used for analysis in Ma et al.'s study. As recommended by the Prostate Imaging Reporting and Data System (PI-RADS), mpMRI sequences are needed for prostate lesion identification. A recently published study also showed that mpMRI data is more helpful than single sequence data in radiomics analysis for prostate cancers (20). But the potential of multiple MR sequences and a combination with clinical variables to predict EPE have not been fully explored yet.

Thus, this study was designed to develop and validate a radiomics model based on mpMRI for the preoperative prediction of EPE in patients with PCa.

## Materials and Methods

### Patients

The Institutional Review Board (IRB) approved this retrospective study and waived the need for written informed consent. Patients with pathologically confirmed prostate cancer who underwent preoperative prostate mpMRI followed by RP between January 2015 to March 2019 at our institution were retrospectively enrolled in this study. The inclusion criteria were as follows: (1) all patients received RP and had confirmed prostate cancer; (2) complete pathological slices were available; (3) prostate mpMRI was performed within 4 weeks before RP. The exclusion criteria were as follows: (1) complete pathological slices were not available for EPE evaluation (*n* = 2); (2) the patients received a biopsy within 6 months before MRI or received prior therapies (such as radiation therapy and hormonal therapy) before MRI (*n* = 14); (3) the quality of the MR images was not satisfactory with severe motion artifacts (*n* = 0). Supplementary Figure 1 shows a flowchart of patient recruitment in this study, and 95 patients were enrolled in this study.

The clinicopathologic data including age, total prostate-specific antigen (t-PSA) level, free-PSA (f-PSA) level, free/total PSA (F/T), and Gleason group for each patient were obtained from the medical records, and PI-RADS category was assessed by radiologists.

### MR Data Acquisition

A 3.0-T MRI scanner (GE750, GE Healthcare, Milwaukee, WI, USA) with an abdominal eight-channel surface phased array coil was used to perform prostate mpMRI, including T2-weighted imaging (T2WI), diffusion-weighted imaging (DWI), and dynamic contrast-enhanced (DCE) imaging. Corresponding apparent diffusion coefficient (ADC) maps were calculated automatically (using b values of 0, 800 mm^2^/s). The detailed MR imaging acquisition parameters applied in this study are shown in Supplementary Table 1.

### Standard of References

The final histopathologic assessment was defined as the standard reference. One senior pathologist (Y.X., with more than 10 years of experience in prostate specimen interpretation) who was blinded to the MRI reports reviewed all the RP pathological slices (with a whole-mount slice thickness of 0.4 cm). The EPE status for each lesion was recorded by the pathologist. EPE was defined as the presence of prostate tumors extending out of the confines of the prostate (22).

Two radiologists with different experiences in interpreting prostate MRI (G.Z., with 5 years of experience, and H.S., with 13 years of experience) who were blinded to the pathological results reviewed the MRI images according to PI-RADS V2 and recorded the PI-RADS category for each lesion. One dedicated urologist (W.Y.) coordinated the workflow to ensure the tumor depicted by the pathologist matched the lesion analyzed by the radiologist. Only lesions that were visible on MRI images were finally enrolled in subsequent analysis.

### Radiomics Analysis

**Figure 1:**
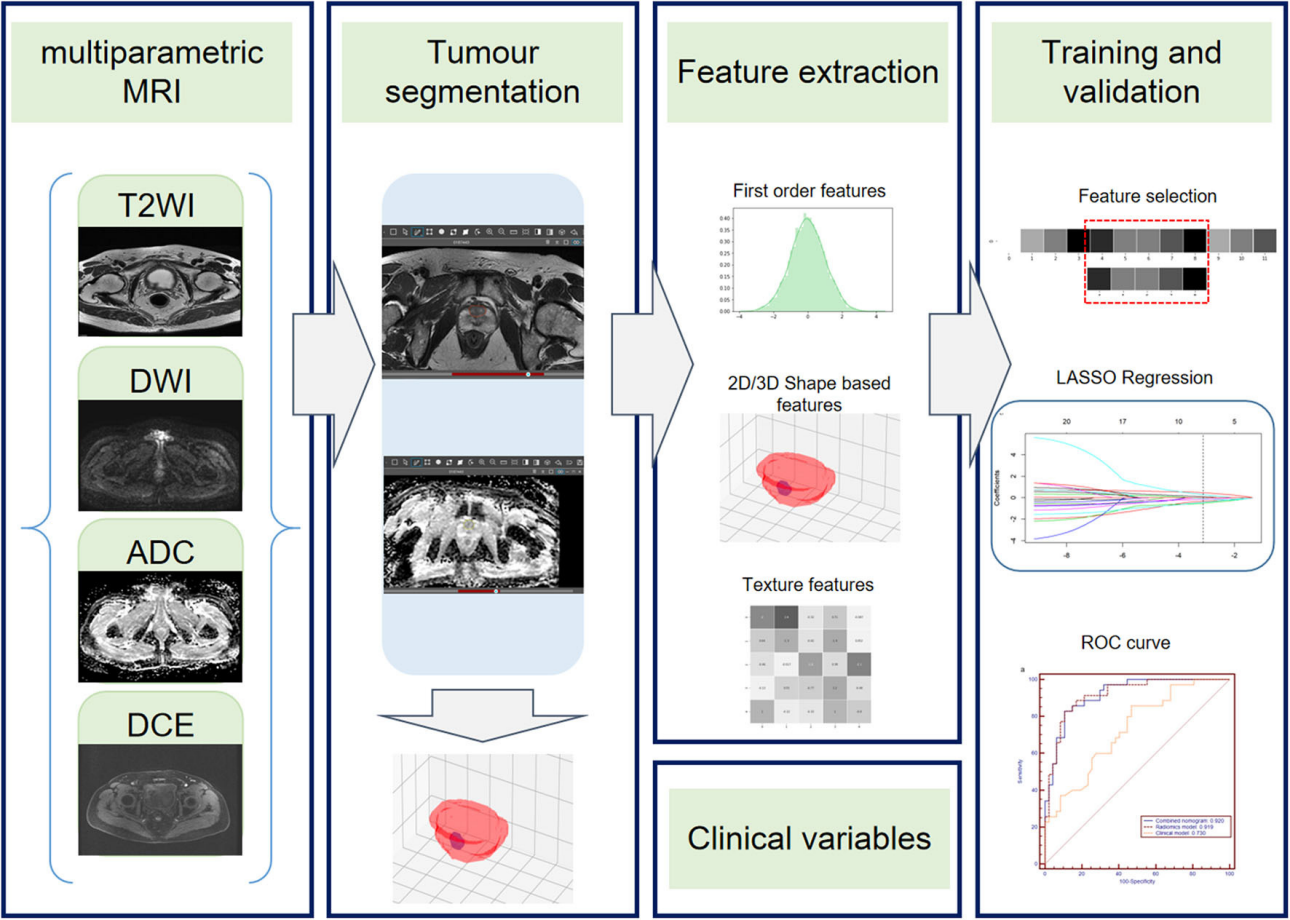
Radiomics workflow. The radiomics workflow includes tumor segmentation, feature extraction, radiomics model, clinical model, and radiomics nomogram construction and predictive performance validation.

The radiomics workflow contained four steps: (1) tumor segmentation; (2) radiomics feature extraction; (3) radiomics feature selection and radiomics model construction; and (4) clinical model and combined nomogram building (Figure 1).

First, one radiologist (G.Z.) who was blinded to the pathologic EPE status performed whole tumor segmentation manually on MR images using Deepwise Research Platform (Deepwise Inc., Beijing, China, http://label.deepwise.com) and a senior radiologist (H.S.) reviewed all the lesions. Two radiologists negotiated to reach an agreement for controversial cases. The senior genitourinary radiologist also delineated half of the lesions to evaluate the inter-class correlation coefficient (ICC). Axial T2WI, DWI, ADC, and DCE images were displayed simultaneously and segmented, respectively, and high *b*-value DWI images were chosen for segmentation. For DCE images, the radiomics features were extracted from the 50 s after the early enhancement phase (23), to make sure that all lesions can be as clear as possible.

Feature extraction was performed using the python package Pyradiomics (version 2.2.0) (24) after ROI was manually segmented. The extraction process was performed using the following three steps: (1) spacing standardization, (2) image filtering, and (3) feature calculation. Thus, a total of 4,580 radiomics features (1,145 features for each sequence) were extracted from the ROI of T2WI, DWI, ADC, and DCE sequences for each lesion. For each MRI sequence, 14 morphology features, 16 intensity-based statistical features, 2 intensity histogram features, 23 gray level co-occurrence matrix (GLCM) features, 14 neighboring gray level dependence matrix (NGLDM) features, 16 gray level run length matrix (GLRLM) features, and 16 gray level size zone matrix (GLSZM) features were calculated on the base images; 64 intensity-based statistical features, 8 intensity histogram features, 92 GLCM features, 56 NGLDM features, 64 GLRLM features, and 64 GLSZM features were calculated on the Laplacian of Gaussian (LoG)-filtered images; 128 intensity-based statistical features, 16 intensity histogram features, 184 GLCM features, 112 NGLDM features, 128 GLRLM features, and 128 GLSZM features were calculated on the wavelet-filtered images.

All lesions were randomly divided into the training group (82 lesions, 35 EPE, and 47 non-EPE) and the validation group (33 lesions, 14 EPE, and 19 non-EPE). The radiomics signatures extracted from DWI, ADC, T2WI, and DCE were combined to build a radiomics model. A maximum relevance minimum redundancy (mRMR) algorithm was applied to assess the relevance and redundancy for each feature. Finally, the 30 highest mRMR-ranked features were retained. Then the least absolute shrinkage and selection operator (LASSO) regression algorithm was conducted to choose the optimized subset of features to construct the final model, and the Rad-score for each lesion was then calculated. The calibration curve and the decision curve of the radiomics model were plotted to analyze the calibration and clinical usefulness of the model. A detailed description of the radiomics analysis process is presented in Supplementary Data 1.

The clinicopathologic factors were first evaluated by univariate analysis. Then, the clinical model was constructed by incorporating the significant clinical features in univariate analysis into a binary logistics regression model. The radiomics model and selected clinical features were integrated to build a combined nomogram using a logistic regression algorithm.

### Statistical Analysis

The differences in the clinicopathological variables between the EPE positive and EPE negative groups were assessed using Student's *t*-test, Mann–Whitney *U*-test, chi-squared test, or Fisher's exact test, where appropriate. The receiver operating characteristic (ROC) curves of the radiomics model, clinical model and the combined nomogram in both the training and validation groups were plotted, and the diagnostic accuracy, sensitivity, and specificity were calculated to evaluate the diagnostic performance of these models. The DeLong test was used to compare the AUCs of different models. The software used for analyses included SPSS 22.0 (IBM, Armonk, NY), MedCalc 11.4.2.0 (MedCalc, Ostend, Belgium), R 3.5.1 (Comprehensive R Archive Network, www.r-project.org), and Python 3.6.0 (Python Software Foundation, Beaverton, OR). A two-tailed *P* < 0.05 was indicative of statistical significance.

## Results

### Clinicopathological Data

In total, 95 patients with 115 lesions (mean age, 64.83 ± 5.39 years; age range, 43–80 years, 66 EPE-negative, and 49 EPE-positive) were enrolled in this study. The clinicopathological data of the lesions in the EPE positive and EPE negative groups are summarized in Table 1. In the training group, in terms of age and F/T, no significant difference was noted between the two groups (*P* > 0.05). However, the t-PSA, f-PSA, PI-RADS category, and Gleason group were significantly different between EPE positive and negative groups (*P* < 0.05). While in the validation group, no significant difference was noted among all clinicopathological variables between the two groups (*P* > 0.05).

**Table 1:** Clinicopathological data of patients in this study.

**Clinicopathological data**	**Per-lesion (*n* = 115)**	**Training group** **(*****n*** **=** **82)**		**Validation group** **(*****n*** **=** **33)**	
		**EPE negative** **(*n* = 47)**	**EPE positive** **(*n* = 35)**	**EPE negative** **(*n* = 19)**	**EPE positive** **(*n* = 14)**
Age (year), mean ± SD	64.83 ± 5.39	64.28 ± 5.32	65.54 ± 5.56	64.16 ± 5.85	65.79 ± 4.76
t-PSA (ng/mL), mean ± SD	13.00 ± 10.54	11.14 ± 6.34	19.16 ± 15.52^*^	8.01 ± 3.36	10.58 ± 5.33
f-PSA (ng/mL), mean ± SD	1.59 ± 1.24	1.41 ± 9.44	2.19 ± 1.63^*^	1.08 ± 0.70	1.40 ± 0.81
F/T, mean ± SD	0.13 ± 0.07	0.13 ± 0.06	0.13 ± 0.06	0.14 ± 0.06	0.16 ± 0.13
**PI-RADS category**, ***n*** **(%)**					
1–2	9 (7.8)	4 (8.5)	1 (2.9)^*^	3 (15.8)	1 (7.1)
3	4 (3.5)	2 (4.3)	1 (2.9)	1 (5.3)	0 (0.0)
4	59 (51.3)	30 (63.8)	11 (31.4)	12 (63.2)	6 (42.9)
5	43 (37.4)	11 (23.4)	22 (62.9)	3 (15.8)	7 (50.0)
**Gleason group**, ***n*** **(%)**					
1	50 (43.5)	22 (46.8)	12 (34.3)^*^	13 (68.4)	3 (21.4)
2	29 (25.2)	14 (29.8)	6 (17.1)	2 (10.5)	7 (50.0)
3	14 (12.2)	6 (12.8)	6 (17.1)	1 (5.3)	1 (7.1)
4	9 (7.8)	1 (2.1)	6 (17.1)	2 (10.5)	0 (0.0)
5	13 (11.3)	4 (8.5)	5 (14.3)	1 (5.3)	3 (21.4)

EPE, extraprostatic extension; t-PSA, total prostate-specific antigen; f-PSA, free prostate-specific antigen; F/T, free/total PSA; PI-RADS, prostate imaging reporting and data system.

* *P < 0.05. Compared by Student's t-test, Mann–Whitney U-test, chi-squared test or Fisher's exact test when appropriate*.

### Radiomics Model Construction

**Figure 2:**
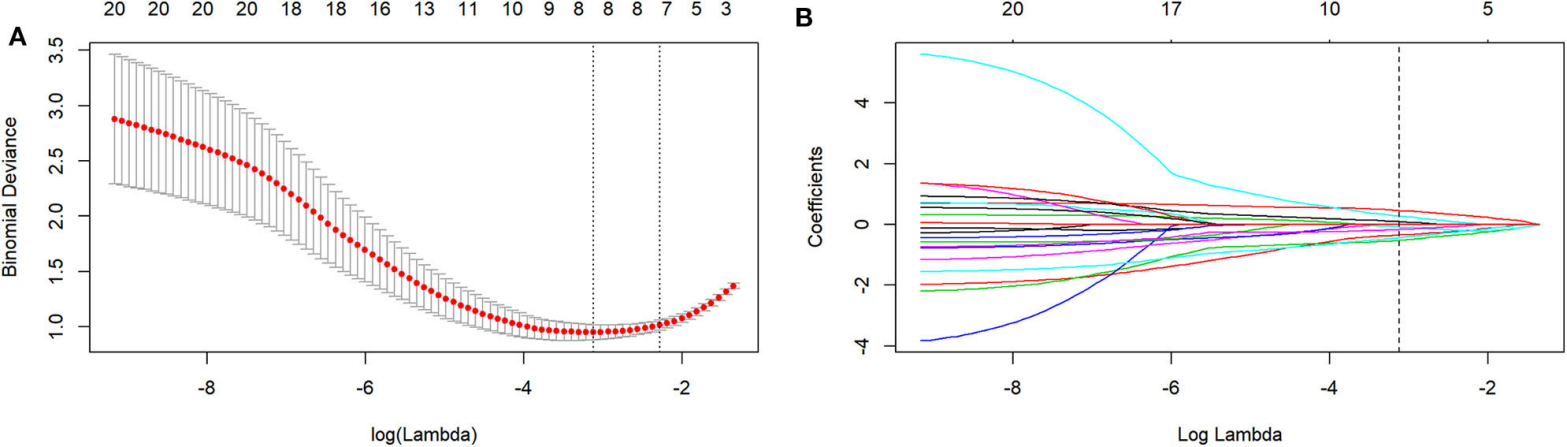
The LASSO includes choosing the regular parameter lambda (λ) **(A)**, determining the number of the feature **(B)**. The optimal λ-value was 0.044237207 with transformed log (λ) of −3.5. Eight features were finally selected.

**Figure 3:**
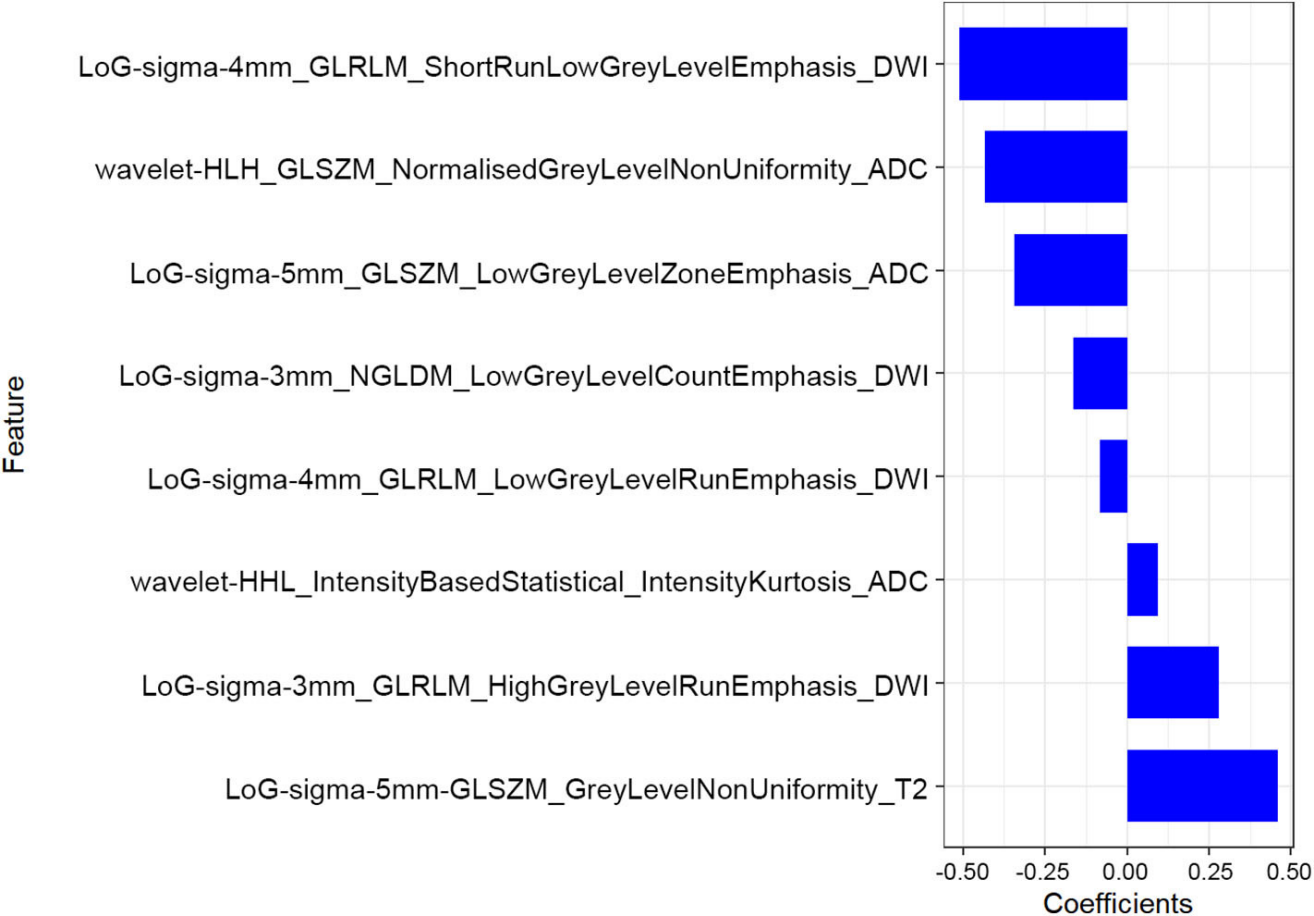
The corresponding coefficients of the most predictive subset of features.

The mean ICC value for the radiomics features was 0.801 (95% CI: 0.612–0.881). Eight radiomics features (1 Intensity Kurtosis from ADC, and 7 texture features [3 GLRLM features from DWI, 2 GLSZM features from ADC, 1 GLSZM feature from T2, and 1 NGLDM feature from DWI]) were selected to build the radiomics model using the LASSO regression analysis (Figures 2, 3). The Rad-score can be calculated as follows: Rad-score = −0.512 × LoGsigma4mm_GLRLM_ShortRunLowGrayLevelEmphasis_DWI-0.433 × waveletHLH_GLSZM_NormalizedGrayLevelNonUniformity_ADC-0.345 × LoGsigma5mm_GLSZM_LowGrayLevelZoneEmphasis_ADC-0.162 × LoGsigma3mm_NGLDM_LowGrayLevelCountEmphasis-DWI-0.082 × LoGsigma4mm_GLRLM_LowGrayLevelRunEmphasis_DWI + 0.093 × waveletHHL_IntensityBasedStatistical_IntensityKurtosis_ADC + 0.281 × LoGsigma3mm_GLRLM_HighGrayLevelRunEmphasis_DWI+0.459 × LoGsigma5mm_GLSZM_GrayLevelNonUniformity_T2-0.452.

**Figure 4:**
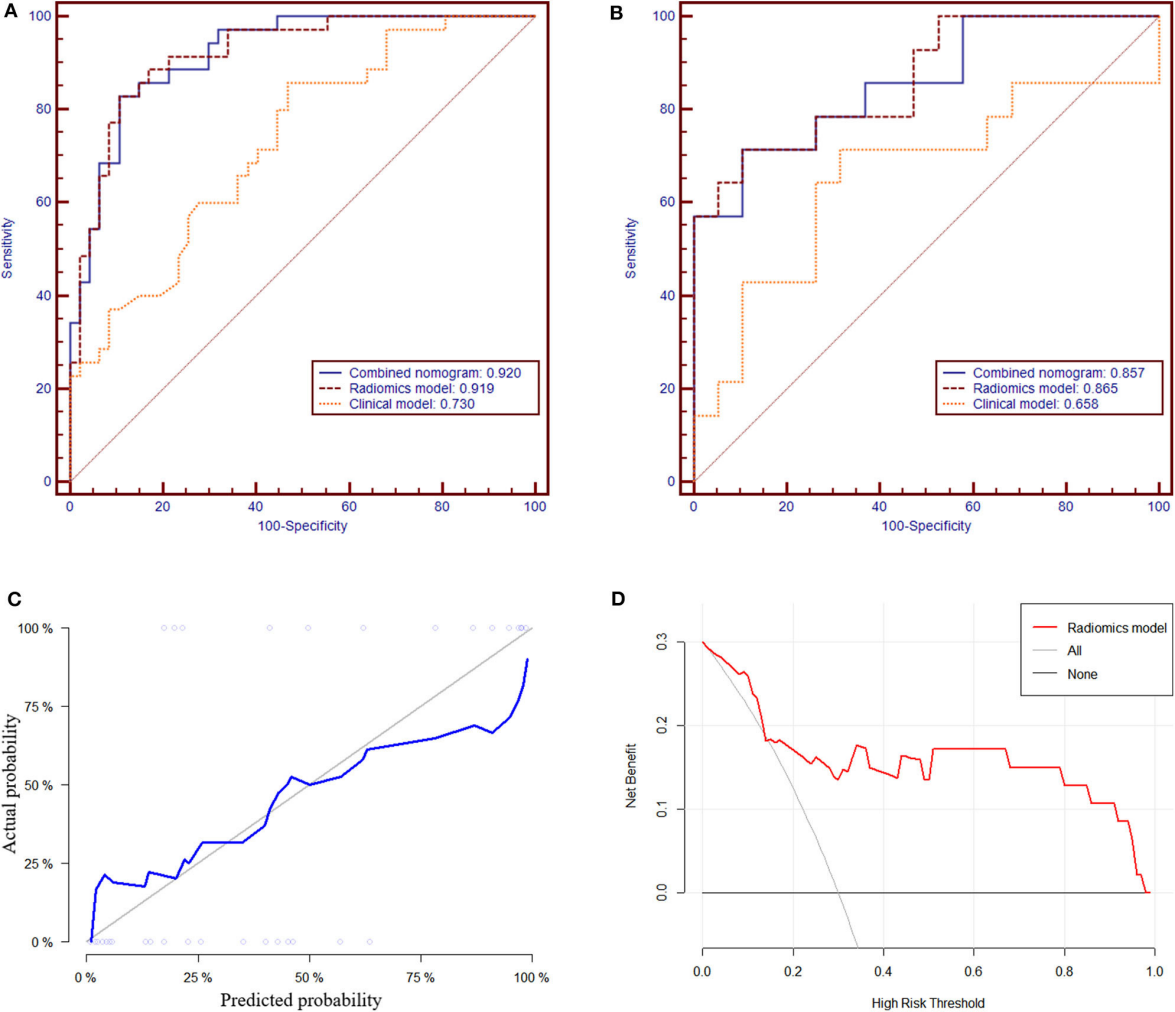
The receiver operating characteristic curves of the radiomics model to differentiate EPE and non-EPE lesions in the training **(A)** and validation group **(B)**. The calibration curve **(C)** of the radiomics model in the validation group showed good agreement between the predicted and actual probabilities. In the decision curve analysis **(D)**, when Pt was 0.15–0.97, the net benefit of the model is better than that of the treat-all or treat-none schemes.

In the training group, the AUC, accuracy, sensitivity, and specificity of the radiomics model were 0.919 (95% CI: 0.861–0.978), 85.4, 82.9, and 89.4%, respectively. In the validation group, the values were 0.865 (95% CI: 0.738–0.992), 81.8, 71.4, and 89.5%, respectively (Table 2 and Figures 4A,B). The calibration curves demonstrated good agreement between the predictive and observation probabilities of EPE and non-EPE lesions for the radiomics model (Figure 4C), and the decision curve indicated the clinical usefulness of this radiomics model in the validation group (Figure 4D).

**Table 2:** Diagnostic performance of different models.

	**Training group**					**Validation group**				
	**AUC**	**AUC 95% CI**	**Accuracy (%)**	**Sensitivity (%)**	**Specificity (%)**	**AUC**	**AUC 95% CI**	**Accuracy (%)**	**Sensitivity (%)**	**Specificity (%)**
Clinical model	0.730	0.622– 0.838	67.1	85.7	53.19	0.658	0.450–0.866	69.7	71.4	68.4
Radiomics model	0.919	0.861– 0.978	85.4	82.9	89.4	0.865	0.738– 0.992	81.8	71.4	89.5
Combined nomogram	0.920	0.863– 0.976	85.4	82.9	89.4	0.857	0.725– 0.989	81.8	71.4	89.5

*CI, confidence interval; AUC, area under the curve*.

### Clinical Model and Combined Nomogram Building

Among the candidate clinical predictors, the t-PSA and Gleason group showed statistical significance (*P* = 0.007 and 0.047, respectively) in logistic analysis and were used to build the clinical model (Supplementary Table 2). The AUC of the clinical model in the training and validation group was 0.730 (95% CI: 0.622–0.838) and 0.658 (95% CI: 0.450–0.866), respectively (Table 2).

The radiomics model and the clinical model were combined to build the radiomics nomogram (Supplementary Figure 2). The AUC of the combined nomogram in the training and validation group was 0.920 (95% CI: 0.863–0.976) and 0.857 (95% CI: 0.725–0.989), respectively (Table 2). In the validation group, the AUC values of the radiomics model and radiomics nomogram were significantly higher than that of the clinical model (*P* = 0.016 and 0.020, respectively). No statistical significance was noted between the AUCs of the radiomics model and radiomics nomogram (*P* = 0.644) in the validation group.

## Discussion

In this study, we built a radiomics model based on mpMRI to predict EPE in patients with prostate cancer. This radiomics model showed satisfactory diagnostic performance for differentiating EPE and showed better performance than that of the clinical model. The combined nomogram showed similar results to diagnose EPE compared with that using the radiomics model alone.

The preoperative prediction of EPE is clinically important for PCa. A randomized trial with a long-term follow up in prostate cancer showed a pathologic EPE rate of 47% (132/283) in localized prostate cancer, indicating that patients with pathologic localized prostate cancer and a long life expectancy may benefit from RP, while the presence of EPE in the radical prostatectomy specimens was highly predictive of death from prostate cancer, with a relative risk of 5.2 (3). Additionally, the presence of EPE in PCa indicated a higher risk of biochemical recurrence after radical therapy (4, 25).

As the standard approach of PCa preoperative assessment, mpMRI has been reported by many studies to diagnose EPE. Krishna et al. (26) assessed the ability of MRI to diagnose EPE using PI-RADS V2, subjective evaluation of EPE, the tumor size, length of capsular contact (LCC), and ADC measurement, with an AUC range of 0.56–0.76; the size, LCC, and ADC entropy improved the sensitivity but reduced the specificity compared with subjective analysis. A Likert score conveying the degree of suspicion at mpMRI was also demonstrated to be a strong predictor of EPE (27, 28). A diagnostic meta-analysis showed that the sensitivity and specificity of mpMRI to diagnose EPE were 0.57 (95% CI: 0.49–0.64) and 0.91 (95% CI: 0.88–0.93), respectively (11). The major limitations of subjective mpMRI evaluation are its poor and heterogeneous sensitivity for local PCa staging, observer dependency and heterogeneity in the definitions of positive and negative results (12). The radiomics model we proposed provided a quantitative and objective method for the evaluation of EPE, which showed fair good diagnostic performance in the validation group with a sensitivity of 71.4% and specificity of 89.5%. This model might complement the insufficient sensitivity of subjective mpMRI evaluation.

Apart from subjective MRI evaluation, some quantitative mpMRI parameters, nomograms or grading systems combining MRI and clinicopathological indicators have been proposed by previous studies. Kim et al. (29) investigated the value of mpMRI for EPE using qualitative and quantitative parameters (such as K-trans, K-ep, and Ve), and the AUC values were 0.944–0.957, respectively. Nevertheless, these models were not further validated. Mehralivand et al. (30) proposed an MRI grading system for pathologic EPE; the results indicated that the clinical features plus MRI grading had the highest diagnostic performance to predict pathologic EPE (AUC, 0.81 vs. 0.77, respectively). Studies have shown that MRI can improve the diagnostic performance of clinical-based models to predict EPE (31, 32). A new PartinMR model incorporating the Partin table and mp-MRI using support vector machine (SVM) analysis was developed by Wang et al. (33) and possessed a higher AUC value than that of the Partin table (0.891 vs. 0.730). The incremental benefit of mpMRI over clinical information indicated that the combination of them may be useful in decision making for PCa patients (34). In our study, the combined nomogram was also demonstrated to outperform the clinical model in diagnosing EPE but was comparable with using radiomics model alone. It was still too early to draw the conclusion that merely using radiomics features would be sufficient to diagnose EPE because the clinical variables analyzed in this study were not sufficient. Thus, further studies are needed to validate this conclusion and modify the proposed model.

Recently, several studies have reported the application of radiomics in the aggressiveness assessment and prognosis prediction of PCa, as well as in the field of EPE diagnosis. Compared with traditional radiologic interpretation, radiomics could provide more information about the tumor that might be correlated with the intratumor heterogeneity (13). Ma et al. (21) constructed a radiomics signature by a LASSO regression algorithm based on T2WI to predict EPE preoperatively, yielding AUCs of 0.902 and 0.883 in the training and validation cohort, respectively. Compared with the radiologists' interpretations (AUC: 0.600–0.697), the radiomics signature was more sensitive but obtained comparable specificity. Stanzione et al. (35) assessed the possibility of machine learning algorithms to predict EPE using texture analysis (TA), features extracted fromT2WI, and ADC maps that turned out to be a feasible tool with an AUC value of 0.88. These two studies and ours supported the value of machine learning in the diagnosis of EPE, and the diagnostic performance of these models and ours seems comparable. Nevertheless, only radiomics features based on T2WI were selected in Ma et al.'s (21) study, and only T2WI and ADC maps were analyzed in Stanzione et al.'s (35) study. Because mpMRI sequences are recommended by PI-RADS for prostate lesion identification, we combined radiomics features from mpMRI sequences to build the radiomics model. Compared with Ma et al.'s (21) study (17 features), our model only used eight features and showed fair good diagnostic performance. Interestingly, only features from T2WI, ADC, and DWI images were used in our model, which indicated that these images might be more helpful than DCE in the assessment of EPE. And most of the selected features were texture features from DWI and ADC sequences. It could be hypothesized that MRI images especially functional images could provide more useful information for the training of radiomics model, and texture features rather than intensity-based statistical features or morphology features could be more helpful in the diagnosis of EPE.

There are several limitations to our study. First, it was a single-center study with a relatively small sample size, without external validation; thus, a larger sample-sized multicenter study is needed for validation in future clinical applications. Second, only images from one scanning machine were used, and how the heterogeneity of different scanners would affect the reproducibility of the radiomics model has not been analyzed. And whether radiomics models based on single MRI sequences would be comparable with the model based on mpMRI has not been analyzed. Finally, some subjective morphologic features of EPE in mpMRI were not included in the nomogram to explore its potential added value. Future studies are ongoing to make the radiomics model a more reliable one.

In conclusion, the radiomics model based on mpMRI could different EPE and non-EPE lesions with satisfactory diagnostic performance, which might be a feasible tool to preoperative predicting EPE and assist in the decision making for the individual treatment of PCa.

## Data Availability Statement

The original contributions presented in the study are included in the article/Supplementary Materials, further inquiries can be directed to the corresponding authors.

## Ethics Statement

The studies involving human participants were reviewed and approved by Institutional Review Board of Peking Union Medical College Hospital. Written informed consent for participation was not required for this study in accordance with the national legislation and the institutional requirements.

## Author Contributions

Guarantor of the article: ZJ and HS. Conception and design: HS, JL, and XL. Collection and assembly of data: GZ, HS, LM, XL, WY, and YX. Data analysis and interpretation: LX, GZ, and LZ. Manuscript writing and final approval of manuscript: All authors.

## Conflict of Interest

LZ, LM, and XL were employed by the company Deepwise AI Lab, Deepwise Inc. The remaining authors declare that the research was conducted in the absence of any commercial or financial relationships that could be construed as a potential conflict of interest.
