# Supplementary material for: Radiomics Based on Multiparametric Magnetic Resonance Imaging to Predict Extraprostatic Extension of Prostate Cancer
Source: Front Oncol. 2020 Jun 16;10:940. doi: 10.3389/fonc.2020.00940 (PMC7308458; doi:10.3389/fonc.2020.00940)

Supplementary Material

**1 Supplementary Data 1:** The detailed description of radiomics analysis process

**Radiomics features extraction:** The extraction process was done following three steps:

1) Spacing standardization: All images were resampled using interpolator of sitkBSpline in Python package SimpleITK to pixel spacing of 1.0 mm in all three dimensions, to exclude the disturbance caused by various scales.

2) Image filtering: Wavelet filter and Laplace of Gaussian filter with different sigma were applied to the original images to enhance discrimination of features. Specifically, the wavelet filter applied a high or a low pass filter in each dimension, thus 8 decompositions per level were achieved. Laplace of Gaussian filter emphasized coarser texture with higher sigma and finer texture with lower sigma. These filtered images are used to calculate features aside from original unfiltered images.

3) Feature calculation: Predefined statistics features were calculated. First-order statistics describing the distribution of voxel intensities within the image region defined by the mask, shape-based metrics describing the three-dimensional size and shape of the ROI which are independent of the gray level intensity distribution in the ROI. Gray Level Co-occurrence Matrix (GLCM), Gray Level Run Length Matrix (GLRLM), Gray Level Size Zone Matrix (GLSZM), Gray Level Dependence Matrix (GLDM) are all metrics describing the texture inside the ROI. A total of 1145 radiomics features were extracted from the ROI of a single sequence for each lesion.

**Radiomics features selection and radiomics model construction:**

The maximum relevance minimum redundancy (mRMR) algorithm was used to assess the relevance and redundancy for each feature. The maximum-relevance selection aimed to select features that were highly correlated to the EPE positive status. The minimum-redundancy selection filtered features that were highly redundant with each other. The features were ranked according to their relevance-redundancy indexes in mRMR analysis. The top 30 features with high-relevance and low-redundancy were used to construct the radiomics model using the least absolute shrinkage and selection operator (LASSO) algorithm.

**2 Supplementary Table 1: Sequence parameters for prostate multiparametric MRI**

| **Parameters** | **T2WI** | **DWI** | **DCE** |
| --- | --- | --- | --- |
| Sequence | FRFSE | SE-EPI | 3D-GRE |
| TR/TE (ms) | 4137/86 | 4200/90 | 4.3/1.3 |
| Flip angle (degree) | 110 | 90 | 12 |
| Echo train length | 32 | 1 | N/A |
| Field of view (mm × mm) | 270 × 270 | 360 × 360 | 400 × 400 |
| Matrix size | 288 × 192 | 128 × 96 | 320 × 192 |
| Thickness (mm) | 3.0 | 3.0 | 3.0 |
| Other |  | b values= 100, 150, 200, 500, 800, 1000, 1500, 2000 mm^2^/sec | Temporal resolution <10s, and total scan time of 5 min |

*TR, repetition time; TE, time echo; FRFSE, fast relaxation fast spin echo; SE-EPI, spin-echo echo planar imaging; 3D-GRE, 3D-gradient echo; DWI, diffusion-weighted imaging; T2WI, T2-weighted imaging; DCE, dynamic contrast-enhanced; ADC, apparent diffusion coefficient; DCE images were obtained with one dose of gadopentetate dimeglumine (Magnevist; Bayer Healthcare) at a dose of 0.1 mmol per kilogram of body weight administered through a peripheral vein at a rate 3mL/sec by using an automatic injector (Spectris Solaris EP; Medrad).*

**3 Supplementary Table 2:** Logistic regression analysis of clinical features

| Clinical features | β | SE | Wald | *P* | Odds ratio (95% CI) |
| --- | --- | --- | --- | --- | --- |
| t-PSA | 0.099 | 0.037 | 7.372 | 0.007 | 1.104 (1.028-1.186) |
| Gleason group | 0.360 | 0.181 | 3.941 | 0.047 | 1.434 (1.005-2.046) |

*t-PSA, total prostate specific antigen; CI, confidence interval.*

**4 Supplementary Figure 1:** Flowchart for patient enrollment


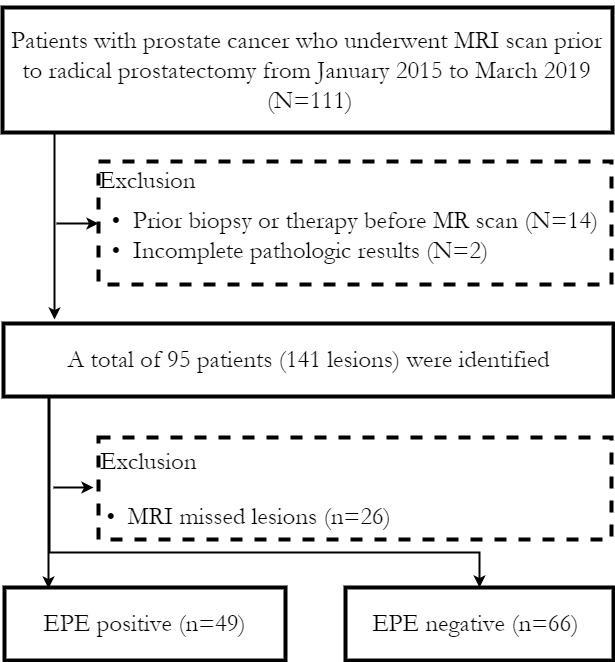


**5 Supplementary Figure 2:** Figure of the combined nomogram


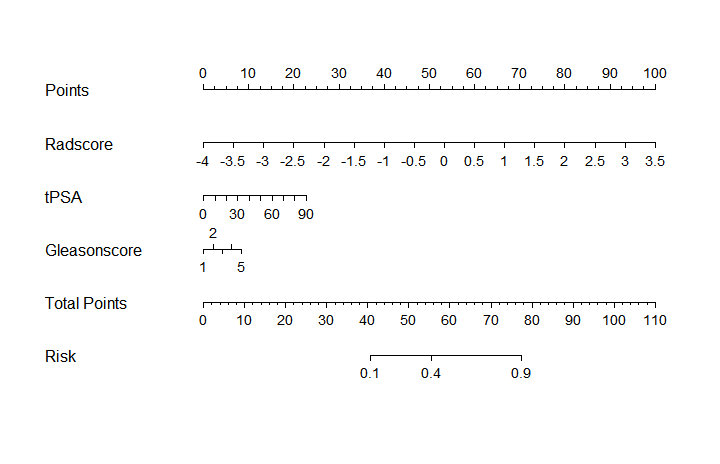

Supplement: Supplementary file 1 [file Data_Sheet_1.DOC]
